# Brief Report - Monoclonal Antibodies Illustrate the Difficulties in Measuring Blocking TSH Receptor Antibodies

**DOI:** 10.3389/fendo.2022.943459

**Published:** 2022-07-15

**Authors:** Terry F. Davies, Syed A. Morshed, Mihaly Mezei, Rauf Latif

**Affiliations:** Thyroid Research Unit, Department of Medicine, Icahn School of Medicine at Mount Sinai and James J. Peters VA Medical Center, New York, NY, United States

**Keywords:** TSH receptor (TSHR), Graves’ disease, thyroid stimulating antibodies, thyroid blocking antibodies, thyroid bioassay, Hashimoto autoimmune thyroiditis

## Abstract

TSH receptor (TSHR) antibodies are the cause of Graves’ disease and may also be found in patients with Hashimoto’s thyroiditis. They come in at least three varieties: thyroid stimulating, thyroid blocking and neutral. The measurement of TSH receptor antibodies in Graves’ disease and Hashimoto’s thyroiditis is a common clinical activity and can be useful in diagnosis and prognosis. We show that it is not possible to detect the blocking variety of TSHR antibody in patients with Graves’ disease because the stimulating antibody may overwhelm the measurement of blocking in the bioassays available for their measurement and may blind the valid interpretation of the results. To help explain this in more detail we show a series of studies with monoclonal TSHR antibodies which support this conclusion.

## Introduction

Graves’ disease was once thought to be secondary to excess TSH from the pituitary but the discovery of thyroid stimulating antibodies, or Long Acting Thyroid Stimulator (LATS) as it was first known, by Adams and Purves, in the serum of patients with the Graves’ Disease Triad (hyperthyroidism, orbitopathy and dermopathy) began the modern understanding of Graves’ disease autoimmunity (1, 2). In the beginning, the *in vivo* guinea pig and mouse bioassays used radioactive iodine to look at uptake into the thyroid or discharge from the thyroid gland and compared TSH preparations with patient sera (3). In a classic report, Adams injected himself and his colleagues with serum from a Graves’ patient and showed that their thyroid hormone level increased (2). Once TSH could be radiolabeled without losing its biological activity, it became possible to rapidly detect such stimulating activity in sera from patients with Graves’ disease since the antibody had been shown to be an immunoglobulin (IgG) which would compete with TSH for binding to the TSHR. Subsequently, a series of TSH receptor assays, using TSH binding inhibition, were introduced, first by Smith and Hall (4), which used thyroid membranes or solubilized receptors. These have been improved further as automated, protein-binding, capture immunoassays (5). Although these binding assays are cheap, rapid and easy they only measure the actual antibody levels and do not give the bioactivity associated with the antibodies. Therefore, to avoid the complex rodent assays, cell based systems have been used where the aim is to measure the intrinsic biological activity compared to TSH (6, 7). A large literature shows that the vast majority of patients with new onset and untreated Graves’ disease have detectable TSHR-Abs (over 90%) making their measurement a useful clinical diagnostic tool.

We now understand that TSH receptor (TSHR) antibodies may come in a variety of forms with differing biological activity (8). They may be thyroid stimulating, thyroid blocking, or they may be neutral in relation to TSH signaling but have stress effects on the thyroid cells (9–11). However, the introduction of multiple types of assays can be confusing. We have tried to simplify this situation by carrying out a series of studies using highly specific monoclonal TSHR antibodies and detected their TSHR stimulating and blocking activity patterns and their interactions.

## Methods

### Detecting TSHR Stimulating Activity

We used a previously published transcriptional-based luciferase assay for measurement of TSH and TSH-like bioactivity intrinsic to stimulating TSHR antibodies by measuring an increase in cAMP activity (the TSHR- assay) (7). Briefly, all measurements were carried out in 348 flat bottom white micro titer plates seeded with 15,000 cells per well in complete Ham’s F12 cell culture medium and incubated at 37°C overnight. For measuring stimulating activity, the 35µl of the pre-diluted antibody/TSH in the stipulated concentrations were diluted in serum free F12 medium and added to triplicate wells of the plate after completely emptying the wells and gently tapping the plate on absorbent paper. After addition of the stimulant, the plates were further incubated for 4hrs in a >90% humid chamber at 37°C, following which 13µl of luciferase substrate containing the lysis buffer (BrightGlo- from Promega Inc) was added to each well and incubated for 3 minutes at room temperature on a rocking shaker and finally the plates were measured for luminescence using a ClarioStar microplate reader.

### Detection of TSHR Blocking Activity

For measurement of blocking activity of TSHR antibodies the same bioassay was used with modifications. Cells were pre-incubated with 35µl of known concentrations of the blocking antibody for 30 minutes at 37°C which was followed by addition of 35µl of a fixed concentration of 40µU/ml of pre diluted bovine TSH in all the required wells in triplicate. As before, plates were incubated further for 4hr at 37°C in humid chamber and followed by subsequent steps similar to that described above. For controls we used medium alone as background and wells that had TSH or known stimulating or blocking antibody were used as positive controls. All measurements were performed in 3 independent experiments. Mean and standard deviations were calculated from these experiments using Microsoft Excel and data reduced to represent % stimulation or % inhibition of TSH or stimulating antibody activity. The data were graphically represented using GraphPad Prism software.

### Monoclonal Antibodies Used

We used 4 highly specific TSHR monoclonal antibodies (mAbs) (**Table 1**). We included a highly potent stimulating TSHR-mAb (M22) (Kronus Inc. Star, ID) (12) and our less potent stimulating TSHR-mAb (MS-1) (13). We also included a highly potent blocking TSHR-mAb (KI-70) (gift from RSR Inc, Cardif, UK) (14) and one of our less potent blocking TSHR mAb (TAb-8) (15).

**Table 1:** Monoclonal antibodies to the TSH receptor used in the described studies.

Name	Origin	Activity	Class	Reference
M22	Human	Strong Stimulator	IgG1-lamda	(12)
MS-1	Hamster	Weaker Stimulator	IgG2	(13)
KI-70	Human	Strong Blocking	IgG1-lamda	(14)
TAb-8	Hamster	Weaker Blocking	IgG2	(15)

### Statistics

All data were analyzed using GraphPad Prism Version 6.04. Mean and SD values were used from triplicate measurements. 1way ANOVA with Bonferroni’s multiple comparison test was applied. P<0.05 was considered statistically significant.

## Results

### Characterization of the TSHR mAbs

**Figure 1:**
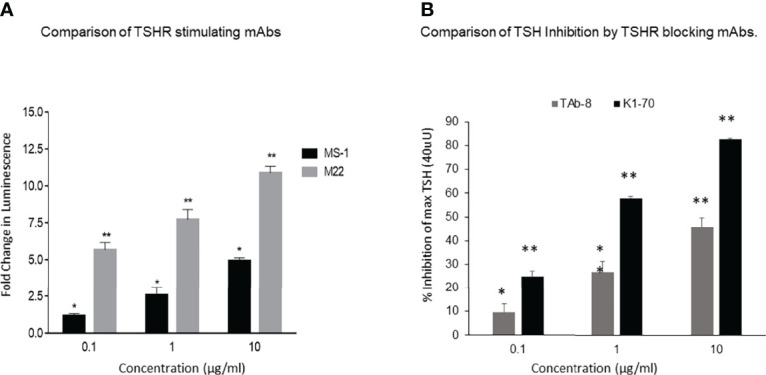
Activation of receptor by stimulating TSHR mAbs with variable potency and inhibition of TSH by strong and weak TSHR blocking mAbs.These and subsequent data was obtained using Chinese Hamster Ovary (CHO) cells transfected with the human TSHR (7). **(A)** Here we show the TSHR stimulating activity of a highly potent (M22) (grey bars) versus a weaker (MS-1) stimulating TSHR mAb (black bars), as measured by deduced cAMP generation in the bioassay. The fold changes of the responses indicated in the y-axis are based on luminescence (luciferase units). These data show the difference in the potency of these two antibodies in stimulating the TSHR and illustrate that in patients with Graves’ disease there is likely to be much variability in the biological activity of the stimulating antibodies even without the possible presence of blocking antibodies. By definition, TSH signaling is inhibited by both highly potent and weaker blocking TSHR antibodies when measured using a TSH bioassay. * = p<0.05, ** = p<0.01 **(B)** A weaker blocking antibody, shown here, was a hamster mAb (TAb-8) which gave ~45% maximum inhibition of TSH stimulation (dark gray bars) at the highest dose tested in the CHO-TSHR cells. In contrast, a stronger human blocking mAb (K1-70) was able to give ~ 85-90% inhibition at the same concentration. We have used these two mAbs throughout the illustrations for easy comparison. * = p<0.05, ** = p<0.01.

**Figure 2:**
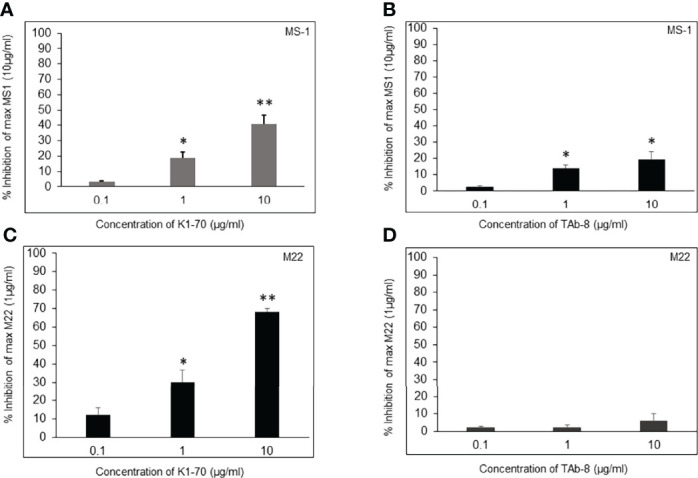
Assessment of two TSHR blocking mAbs in the presence of stimulating mAbs (MS-1 and M22). These bar graphs illustrate how blocking and stimulating TSHR mAbs interact without the influence of TSH. We show Inhibition of a weaker and strong stimulating mAb (MS-1 versus M22) in the presence varying doses of strong and weaker blocking mAbs (K1-70 versus TAb-8). * = p<0.05, ** = p<0.01 **(A, B)** CHO-TSHR cells were stimulated with MS-1 after incubating with increasing doses of K1-70 **(A)** or TAb-8 **(B)** as indicated. After background subtraction the percent inhibition observed to a maximum stimulating dose of MS-1 (10ug/ml) was plotted as % inhibition on the y- axis. The presence of strong blocking antibody caused >40% inhibition whereas in the presence of the weaker blocking antibody the inhibition was <15%. **(C, D)**Here the inhibition measurements were assessed in the presence of the potent stimulating mAb (M22). As shown, much greater inhibition of M22 was obtained by the presence of K1-70 whereas the weaker blocking antibody (TAb-8) showed very poor inhibition against this potent stimulator. These data illustrate how the variable potency of stimulating antibodies in patient serum samples may be influenced by blocking antibodies with different potencies when present in the sample.

Both TSHR stimulators activated the TSHR very effectively (**Figure 1A**) and both blockers inhibited TSH effectively (**Figure 1B**). Both blocking mAbs were also capable of inhibiting stimulating TSHR-Abs (**Figure 2**). However, the higher potency blocker (KI-70) was able to inhibit both weaker and more potent antibodies (**Figures 2A, 2C**) while the weaker blocker (TAb-8) was not able to have a major impact on the more potent M22 stimulator (**Figures 2B, 2D**).

### Difficulties in Measuring Blocking TSHR Antibodies

In patients with Graves’ disease bioassays for TSH stimulation may be performed in the presence of potentially two different antibodies; stimulating and blocking, with both competing for similar binding sites since by definition they inhibit TSH binding. The potency/affinity of the antibodies, therefore, will likely determine the success of their detection. However, clarity cannot come from the use of patient sera because of the presence of the different types of TSHR antibody. However, we found that a less potent blocking TSHR antibody was unable to prevent a highly potent stimulating TSHR antibody having its effect (see **Figure 2D**). This logic makes the measurement of TSHR blocking antibodies in patients with stimulating antibodies totally unpredictable.

### Modeling the Graves’ Serum Situation

**Figure 3:**
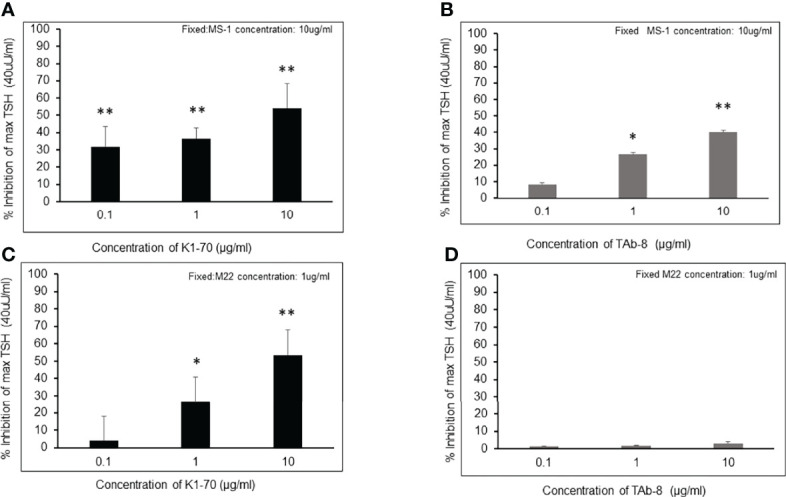
Inhibition of TSH action by a strong and a weaker TSHR blocker in the presence of strong (M22) and less strong (MS-1) TSHR stimulating antibodies These figures try to illustrate the complex real life situation of a serum from a patient with Graves’ disease which contains both stimulating and blocking antibodies and which is added to a TSH bioassay. Since serum cannot be interpreted because of its polyclonality we have mimicked the situation with the four mAbs shown earlier. * = p<0.05, ** = p<0.01 **(A, B)** Inhibition measurements of TSH stimulation were carried out using the weaker stimulating mAb (MS-1) by co-incubating with varying concentrations of potent or less potent blocking mAbs (K1-70 versus TAb-8). Cells were stimulated with a fixed dose of TSH (40 /mL) after incubating with blocking antibody and a fixed concentration of MS-1 (10 /mL). After background subtraction the percent inhibition of TSH stimulation is indicated on the y- axis as in **(A, B)** Good inhibition of TSH was obtained with both of the blocking antibodies when competing with a low potency stimulating antibody. **(C, D)**Here we show inhibition measurements of TSH stimulation carried out using the more potent stimulating mAb (M22) by co-incubating with varying concentrations of strong or weak blocking mAbs (K1-70 versus TAb-8). Cells were again stimulated with a fixed dose of TSH (40 /mL) after incubating with blocking antibody and a fixed concentration of M22 (1/mL). After background subtraction the percent inhibition of TSH stimulation is indicated on the y- axis. The presence of a strong stimulator such as M22 significantly reduced the ability of the low potency-blocking antibody to inhibit TSH **(D)**. If 40% inhibition is considered significant as reported in the literature then TAb-8 was undetectable even at a high concentration.

The observations in **Figure 2** were further illustrated by our studies shown in **Figure 3** with all 3 components present – TSH, stimulating mAb and blocking mAb just as can be expected in a TSH bioassay with certain serum samples from patients with Graves’ disease. The weaker blocking mAb (Tab-8) was unable to inhibit TSH in the presence of a potent stimulating TSHR-mAb (**Figure 3D**) while a highly potent blocking mAb was able to achieve this effect (**Figure 3C**). The logic behind this data shows that blocking TSHR antibodies cannot possibly be reliably detected in a TSH bioassay of serum from patients with Graves’ disease which contain a strong stimulating TSHR antibody. Furthermore, the heterogeneous nature of serum would make the interpretation ambiguous.

## Discussion

The finding that some antibodies to the TSHR which compete for TSH binding but do not initiate normal TSH signaling but rather block TSH induced stimulation identifies the class of TSHR blocking antibodies and mostly reported in a segment of patients with Hashimoto’s thyroiditis (16). In other words, these antibodies bind to the TSHR extracellular domain (ECD) and occupy enough TSH binding sites to prevent TSH ligand binding and thus reduce or inhibit TSH signaling. The fact that a human monoclonal blocking antibody was developed from a patient with Graves’ disease (14) was also proof that such antibodies can be found in patients with Graves’ disease just as stimulating TSHR antibodies may occur in Hashimoto’s thyroiditis (17) where the gland is unable to respond to the stimulation. Indeed, the concept of “Graves’ Alternans” is based on the changing levels/potency of blocking and stimulating TSHR antibodies (16, 18).

In contrast to the multiple assays available for stimulating TSHR antibodies the measurement of TSHR blocking antibodies has remained very unsatisfactory. Their assay is still usually based on bioassays using the inhibition of TSH activating a target cell, usually a TSHR transfected cell, with the read out being either direct cyclic AMP levels or its response elements tagged to luciferase activation as used in this study. In the presence of TSHR blocking antibodies the TSH-induced signal is diminished to a variable degree. By definition, low affinity blocking TSHR antibodies are more difficult to detect than high affinity blocking antibodies because TSH itself is a highly effective thyroid stimulator. This means that in practice only the more powerful blockers may be detected depending upon the assay conditions.

Measuring TSHR blocking antibodies is not usually necessary in clinical practice since it remains unclear how much they contribute to the deterioration in thyroid function of hypothyroid patients. The one situation where the biological assessment of TSHR blocking antibodies may be justified is in pregnancy where neonatal hypothyroidism has been reported secondary to maternal blocking antibody (19) but this has proven to be a very rare occurrence. Hashimoto’s thyroiditis is T cell mediated rather than antibody mediated (20) and the clinically measured thyroid antibodies to thyroglobulin and thyroid peroxidase are secondary to the tissue damage (and hence are polyclonal). However, in such patients measuring blocking TSHR antibodies should be straight forward since they only rarely would have a competing TSHR stimulator present. A reduced TSH signal will indicate the presence of blocking antibodies as reported in up to 20% of such patients (16). Clinically, however, this information is of no major importance but simply adds to our understanding of the thyroid failure. However, since such patients have also been reported to sometimes exhibit stimulating TSHR antibodies, but with less responsive thyroid cells (17) so that even in this situation the measurement of blockers may be unreliable. In clinical practice, TSHR antibodies in hypothyroid patients can also be detected by routine TSH binding inhibition assays, as employed in Graves’ disease, but are likely to be mostly TSHR blocking antibodies.

The problem remains that it is difficult to detect TSHR blocking antibodies in patients with Graves’ disease since their affinity for the TSHR must be greater than the stimulating antibodies causing the hyperthyroidism. This was well illustrated in our studies with monoclonal antibodies where only the most potent blocker could be reliably seen in the presence of a potent stimulator (as illustrated in **Table 2**). Attempts to circumvent this problem have used a series of dilutions (18) but this approach is also dependent on the potency of the different antibodies present. Nevertheless, it has been possible in selected cases to dilute out the stimulating activity leaving a still detectable high potency blocking TSHR antibody.

**Table 2:** This chart illustrates the hypothetical end result of stimulating and blocking TSHR mAbs being present together.

POTENCY	Stimulator	Blocker	Effect	Figure #
high *	+++	+++	Low Block	3B
**mixed**	+++	+	High Activation	3D
**mixed**	+	+++	High Block	3A
**low**	+	+	Low Activation	3C

*As an example, the resulting biological activity will depend on the potency of the competing antibodies. In this case the strong stimulator is better than the strong blocker so the result is just weakened stimulation due to a low degree of blockade.

Note that as shown in the Table and the corresponding Figures it is clear that a weak blocker cannot be easily detected in the presence of a thyroid stimulating antibody.+++ = HIGH + = LOW.

In summary, although the clinical relevance of measuring TSHR antibodies is well established as both an adjunct for the confirmation of a clinical diagnosis of Graves’ disease and helpful in prediction of the disease course, the techniques for measurement of these autoantibodies by clinical laboratories may be confusing. The data presented here illustrates the complexity of the situation in a simple way by using monoclonal TSHR antibodies of the blocking and stimulating type and the interference these may play in TSH bioassays. One major message from these studies is that we cannot easily and reliably detect TSHR blocking antibodies in patients with Graves’ disease using currently available techniques and bioassays.

## Data Availability Statement

The original contributions presented in the study are included in the article/supplementary materials. Further inquiries can be directed to the corresponding author.

## Author Contributions

TD conceived the work, interpreted the data, and wrote the manuscript. SM performed experimental studies and edited the manuscript. MM helped write and edit the manuscript. RL performed experimental studies, prepared the figures, and helped write the manuscript. All authors contributed to the article and approved the submitted version.

## Funding

Funded in part by a VA Merit Award to TD (2 I01 BX000800-09) and generous anonymous philanthropic support.

## Conflict of Interest

TD is a Board Member of Kronus Inc, Star, ID which distributes diagnostics including for TSH receptor antibodies.

The remaining authors declare that the research was conducted in the absence of any commercial or financial relationships that could be construed as a potential conflict of interest.

## Publisher’s Note

All claims expressed in this article are solely those of the authors and do not necessarily represent those of their affiliated organizations, or those of the publisher, the editors and the reviewers. Any product that may be evaluated in this article, or claim that may be made by its manufacturer, is not guaranteed or endorsed by the publisher.
